# Clinical and molecular aspects of human pegiviruses in the interaction host and infectious agent

**DOI:** 10.1186/s12985-022-01769-3

**Published:** 2022-03-09

**Authors:** Mehdi Samadi, Vahid Salimi, Mohammad Reza Haghshenas, Seyed Mohammad Miri, Seyed Reza Mohebbi, Amir Ghaemi

**Affiliations:** 1 Department of Virology, School of Public Health, Tehran University of Medical Sciences, Tehran, Iran; 2 Department of Microbiology, Molecular and Cell-Biology Research Center, Faculty of Medicine, Mazandaran University of Medical Sciences, Sari, Iran; 3 Gastroenterology and Liver Diseases Research Center, Research Institute for Gastroenterology and Liver Diseases, Shahid Beheshti University of Medical Sciences, Tehran, Iran; 4 Department of Virology, Pasteur Institute of Iran, P.O. Box: 1316943551, Tehran, Iran

**Keywords:** HPgV-1, GBV-C, HIV, HCV, Co-infection, Genotype, E2 protein

## Abstract

**Background:**

Human pegivirus 1 (HPgV-1) is a Positive-sense single-stranded RNA (+ ssRNA) virus, discovered in 1995 as a Flaviviridae member, and the closest human virus linked to HCV. In comparison to HCV, HPgV-1 seems to be lymphotropic and connected to the viral group that infects T and B lymphocytes. HPgV-1 infection is not persuasively correlated to any known human disease; nevertheless, multiple studies have reported a connection between chronic HPgV-1 infection and improved survival in HPgV-1/HIV co-infected patients with a delayed and favorable impact on HIV infection development. While the process has not been thoroughly clarified, different mechanisms for these observations have been proposed. HPgV-1 is categorized into seven genotypes and various subtypes. Infection with HPgV-1 is relatively common globally. It can be transferred parenterally, sexually, and through vertical ways, and thereby its co-infection with HIV and HCV is common. In most cases, the clearance of HPgV-1 from the body can be achieved by developing E2 antibodies after infection.

**Main body:**

In this review, we thoroughly discuss the current knowledge and recent advances in understanding distinct epidemiological, molecular, and clinical aspects of HPgV-1.

**Conclusion:**

Due to the unique characteristics of the HPgV-1, so advanced research on HPgV-1, particularly in light of HIV co-infection and other diseases, should be conducted to explore the essential mechanisms of HIV clearance and other viruses and thereby suggest novel strategies for viral therapy in the future.

## Background

It was observed in 1967 that, experimental serum injection of a surgeon named "GB" with acute hepatitis led to a similar disease in tamarins [1, 2]. In 1995, two novel viruses of the Flaviviridae, called GB virus type A and B, were detected in tamarins, which could form hepatitis at the eleventh GB passage following inoculation. These viruses were not able to infect humans [3] nonetheless, a similar human virus (named hepatitis G virus (HGV) or GBV-C) was subsequently identified [4, 5]. In 2010, a less closely related virus (known as GBV-D) was found in bats [6]. Only GBV-B, a 2nd species of the Hepacivirus genus, has been demonstrated to induce liver damage and hepatitis. Infection with this virus can directly lead to acute hepatitis in laboratory animal models such as tamarins [7, 8]. In contrast, GBV-A, HGV/GBV-C not related to hepatitis. In 2011 and regarding genome structure, phylogenetic relationships, and pathogenic features of GBV viruses, it was suggested to categorize GBV-A-like viruses, HGV/GBV-C, and GBV-D as a 4th genus in the Flaviviridae, called Pegivirus (persistent GB virus) [9]. In 2016, following taxonomy updates of the genera Hepacivirus and Pegivirus, a new human pegivirus named HHpgV-1/HPgV-2 was among them. Analysis has revealed more than 94% identity between HHpgV-1 and HPgV-2, suggesting that they are probably the same virus [10–12].

HPgV-1 (formerly GBV-C/HGV) is an enveloped, + ssRNA virus of the Flaviviridae [13], which its genome includes a single open reading frame (ORF) coding structural and non-structural proteins [14, 15]. HPgV-1 shares a sequence homology and genomic structure, with approximately 30% resemblance, to HCV, but its genome is deficient in the E2 hypervariable area and the core sequence [16].

In the same way as other lymphotropic viruses, HPgV-1 is distributed through parenteral and non-parenteral ways and thereby is highly prevalent in communities suffering from a high incidence of certain bloodborne infections and sexually transmitted diseases [13].

HPgV-1 cannot induce any human diseases [17]. So far, there has been no conclusive evidence on the possible relationship between acute or chronic hepatitis and this virus [13, 16, 18]. However, it has positive effects on preventing the replication of HIV-1 and on extending the survivability of Ebola and HIV-1 infected patients [19–21].

Today, HPgV-1 is categorized into seven genotypes and several subtypes according to the genome heterogeneity of their either completely or partially nucleotide sequences. Geographically, such genotypes and subtypes have shown different patterns of dissemination [22].

While HPgV-1 viremia may remain for years, it is ultimately cleared and antibodies against HPgV-1 envelope glycoprotein E2 generally emerge in 50–75% of cases [23].

The existence of HPgV-1 RNA in serum is correlated to active infection, while anti-E2 antibody detection suggests previous infection [24]. Thus, the Anti-E2 antibody is regarded as a valuable indicator for the diagnosis of recovery from HPgV-1 infection, which can be identified after virus clearance [25]. Detection of HPgV-1 is usually done by ELISA and RT-PCR, the two clinical tests detect E2-protein antibodies and HPgV-1 RNA, respectively [26, 27].

In this review, we discuss the history and classification, epidemiology, transmission, life cycle, genome organization, genotypes, immunity, and detection of HPgV-1, as well as its beneficiary impacts, especially in associations and interaction with some other infections.

## Main text

### History and classification

In 1967, during the study of non-A, non-B hepatitis, Dienhardt, and collaborators received a surgeon's serum with acute hepatitis, which induced hepatitis in tamarins. The passage of serum acquired from the injected animals into new animal models showed comparable hepatitis in recently inoculated tamarins as well as in other species of New World monkeys. The effective transmitted agent was named "GB" (the first letters of G. Barker; the surgeon's name) and was widely studied as a presumed agent of non-A, non-B [1, 2].

In 1995, Abbott Laboratories researchers detected two novel viruses in the liver and serum of tamarins acquired hepatitis after inoculation with the eleventh GB agent animal passage. Due to the history of infectious serum, these viruses were called GB virus A and B (GBV-A and GBV-B) [3]. In 1995, the 3rd virus was detected, namely GBV-C, utilising degenerate primers to magnify similar viral sequences in a West African patient with non (A–B) hepatitis [4]. Concurrently, a group of researchers at Genelabs Technologies detected novel sequences of RNA viruses in human serum with chronic non-A, non-B hepatitis, and named it hepatitis G virus (HGV) [5]. Genome analysis of HGV and GBV-C demonstrated that these viruses were small variations of the same virus species [4, 28, 29]. In bats, another more genetically similar virus known as GBV-D was identified [6].

Focused on the nucleotide sequence and genome structure connections, ‘GB’ viruses are members of the Flaviviridae family [30]. Phylogenetic study of the conserved areas of the polymerase sequences and helicase showed that GBV-B and HCV have a close relationship, whereas GBV-A, HGV/GBV-C, and GBV-D create a separated cluster [4].

Although GBV-B, as the real GB-agent, seems not to have been induced by the GB agent and does not contaminate chimpanzees or humans, it induced acute hepatitis in experimentally diseased animals, like intrahepatic transcripts of recombinant GBV-B RNA [8, 31] but, GBV-A described indigenous virus not correlated with hepatitis and several GBV-A-like viruses were consequently detected from New World monkeys [3, 32]. GBV-C has been reported as a natural human virus that has not been linked to hepatitis [4, 13].

**Fig. 1:**
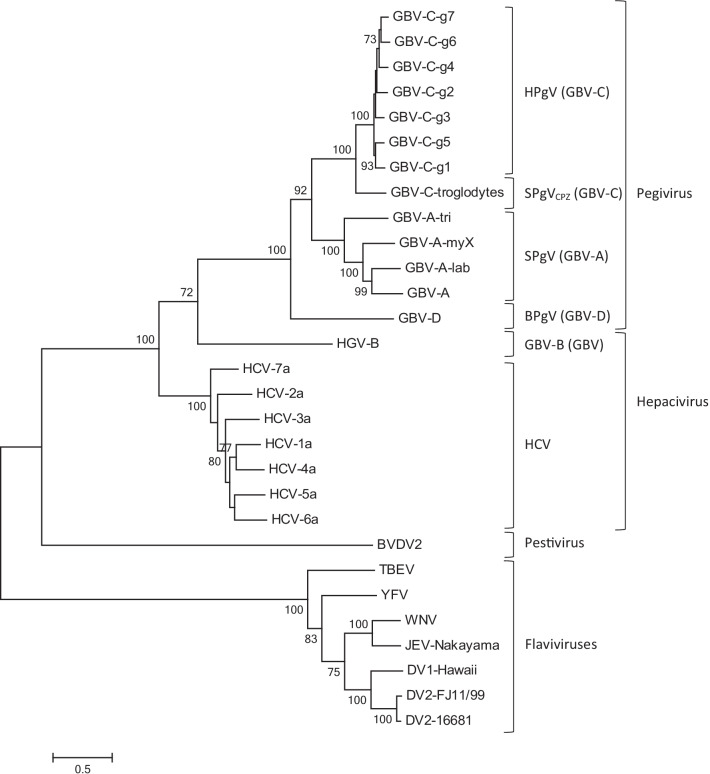
Proposed categorization of GB viruses.Maximum likelihood tree (Tamura 3-parameter nucleotide model and gamma distribution, T92 + G) depicting phylogenetic relationship of Human Pegiviruses with other main members of Flaviviridea family. The phylogenetic tree was constructed based on the complete coding sequence (CDS) of these viruses. The values at the tree branches are the bootstrap support values calculated from 1000 replicates. Scale bar indicates an evolutionary distance of 0.5 substitutions per position in the sequence. GB virus type A, B, C, D (GBV-A, GBV-B, GBV-C, GBV-D), simian Pegivirus(SPgV), human Pegivirus(HPgV), Bat Pegivirus(BPgV), CPZ: chimpanzees

In 2011, The International Committee on Taxonomy of Viruses endorsed the creation of the genus Pegivirus in the Flaviviridae family [9]. GBV-B was the only member that has been described as the second species inside the Hepacivirus genus. According to phylogenetic links, genomic organization, in vivo persistence capabilities, and a clear lack of pathogenicity, it was suggested that GBV-A, GBV-C, and GBV-D should be defined as the 4th genus in the Flaviviridae known as pegivirus (persistent GB viruses) [9, 33]. In addition, it was suggested to rename ‘GB’ viruses to represent their host source inside the preliminary genus Pegivirus. For instance, in this proposed classification, simian Pegivirus (SPgV) would take the place of GBV-A and GBV-A-like viruses and when it comes to species-specific viruses the subscripts would represent the name of the species (SPgV_mys_, SPgV_tri_, SPgV_lab,_ and SpgV_jac_). Moreover, some other conversions would follow as GBV-C/HGV to human Pegivirus (HPgV), GBV-C_cpz_(chimpanzees) to SPgV_cpz_, and GBV-D to bat Pegivirus(BPgV) [9] (Fig. 1).

**Fig. 2:**
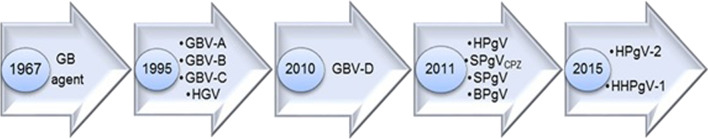
Timeline of discoveries related to pegiviruses

In 2015, a new human pegivirus was separately discovered in the United States and temporarily called human hepegivirus 1 (HHpgV-1) [11] or human pegivirus two (HPgV-2) [10]. The sequences of these viruses shared 94–96% similarity, proving that, they are both the same virus. Although HPgV-2/ HHpgV-1 shares several characteristics with HCV and other hepaciviruses, for example, a core protein, a type iv internal ribosome entry site, and a highly glycosylated E2 protein. HPgV-2/HHpgV-1 phylogenetically is a member of the genus Pegivirus instead of Hepacivirus [10–12, 34] (Fig. 2).

In 2016, Smith et al. suggested a revision of the Pegivirus and Hepacivirus genera taxonomy to include 14 species of hepacivirus and 11 species of pegivirus [33]. In this article, from now on, HPgV (formerly named GBV-C/HGV) will be called HPgV-1 (HPgV type 1) to differentiate from HPgV-2/HHPgV-1.

### Epidemiology

HPgV-1 has been distributed all over the world and is prevalent in the general population. In developed nations, up to around 4% of healthy blood donors possess HPgV-1 viremia [35, 36] and 5–13% of them have anti-E2 antibodies [37–39]. This is while the incidence of HPgV-1 RNA in developing nations reaches 5–18% in the general population [40–43].

Factors involved in HPgV-1 infection include injection of drug use [44], blood and blood products transfusion [13, 45], elevated number of sexual partners [13], sexual intercourse with other men [13, 34], background of sexually transmitted infections, health services, imprisonment [13], medical procedures hospitalization [44, 46], endoscopy, cocaine snoring [13] and travel history to Africa [44].

In general, a high incidence of HPgV-1 has been reported among subjects at risk of parenteral exposure such as those exposed to blood and blood products, hemodialysis, hemophiliacs, chronic hepatitis C or HIV infection, and intravenous drug users. For those people that are at increased danger of other intravenously transmissible infections, including HCV, HIV, or HBV, the frequency of HPgV-1 is around 50% [34, 46, 47].

### Transmission

As with other lymphotropic viruses, HPgV-1 is transmitted sexually, vertically from mother to child during pregnancy and delivery [48] intra-familiarly, intravenous drug use, and by contact with infected blood and blood components [27]. It is therefore very common in people with other blood-borne or sexually transmissible diseases. For instance, the prevalence of HPgV-1 viremia is around 20% among individuals with chronic HCV disease and 20–40% among HIV-positive individuals [13]. Furthermore, particular populations seem especially are prone to the development of HPgV-1 infection, such as those with blood and blood products exposure, Hemophilia people (7–38%), patients requiring hemodialysis (above 10%), and injection drug users [13, 34, 46, 47].

A variety of other non-parenteral modes of infection for HPgV-1 transmission has been identified such as percutaneous contamination from saliva, nosocomial infection through a patient to patient and intrafamilial transmission [13, 49].

Regardless of the risk of parenteral contamination, multiple studies have shown that the prevalence of HPgV-1 infections in both heterosexual and homosexual populations is associated with sexual activity [50] and male to male sex has been described as an efficient form of transmission [22, 34]. Furthermore, sexual transmission of HPgV-1 seems more effective than that of HCV, likely due to lymphotropic and higher concentrations of serum viruses [9].

### The Flaviviridae features and life cycle

**Fig. 3:**
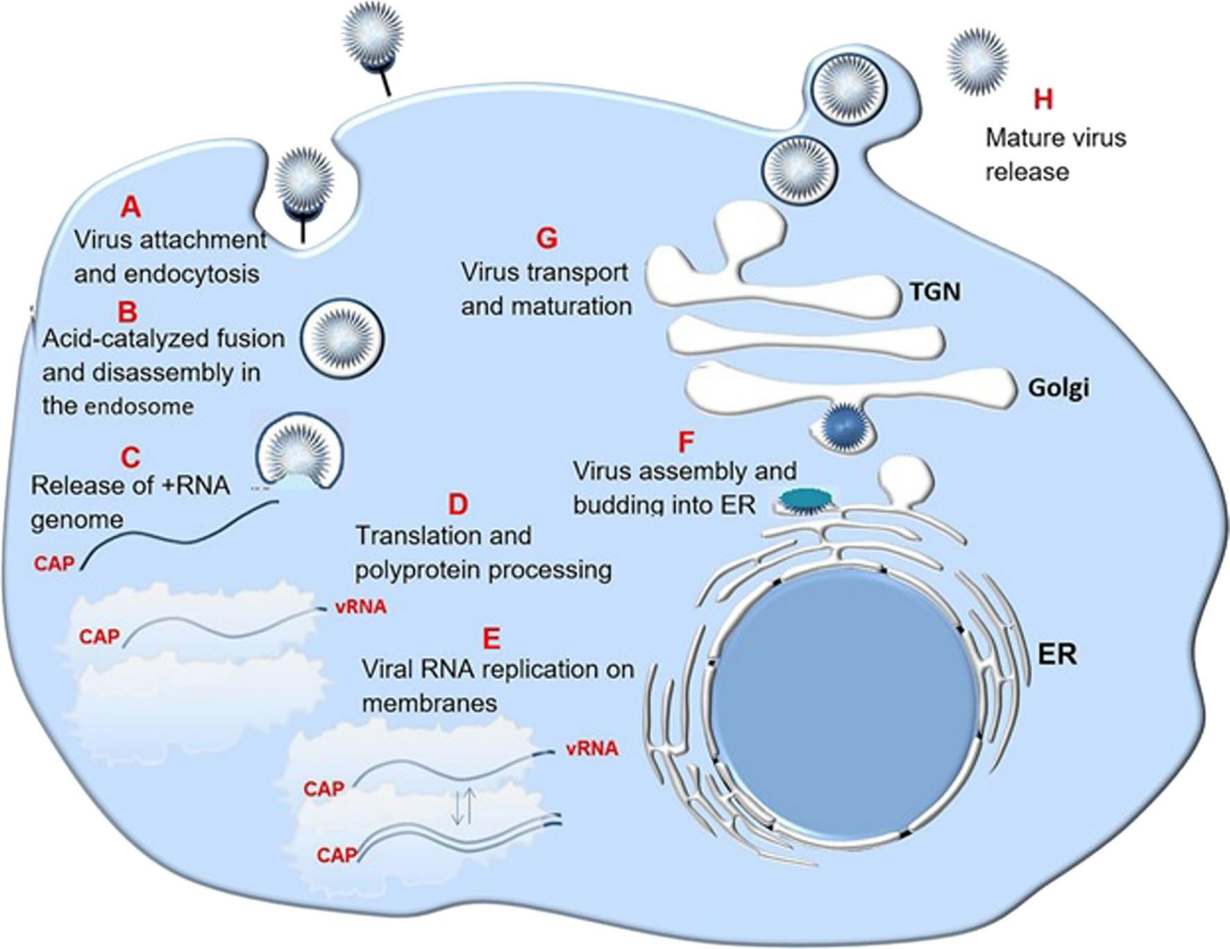
The life cycle of the Flaviviridae. **a** Virus binding and endocytosis mediated by receptor. **b** Acid-catalyzed fusion and disassembly in the endosome. **c** Release of RNA genome into the cytoplasm. **d** Viral RNA translation and polyprotein processing. **e** RNA genome replication on intracellular membranes. **f** Progeny virus assembly and budding into endoplasmic reticulum(ER). **g** Virus transport and maturation through the cell secretory pathway. **h** Virus release at the cell surface

The enveloped virions consist of a lipid membrane comprising two or more envelope (E) glycoprotein species, encompassing a nucleocapsid composed of a + ssRNA genome complexed with several copies of a small, basic capsid (C) protein. Their binding and absorption are thought to include endocytosis mediated by receptors. The low endosome pH causes the virion envelope to be fused with the cell membrane. Following the nucleocapsid uncoating, the genome of RNA is liberated into the cytoplasm. In the lifecycle, the genome has two different functions, one as the template for RNA replication and the other as the mRNA for all viral protein translation. The genome structure is identical across all genera. Viral proteins constitute parts of a single polyprotein, which is broken down by host and viral proteases. RNA replication takes place fully within the host cell in conjunction with the membranes inside the cell. The intermediate RNA is provided by the synthesis of a genome-length minus-strand RNA. Progeny viruses assemble in an internal membrane pocket, most probably the ER, through budding, and after that, they pass via the cell secretory pathway before being discharged at the surface of the cell [51] (Fig. 3).

### Genome organization

**Fig. 4:**
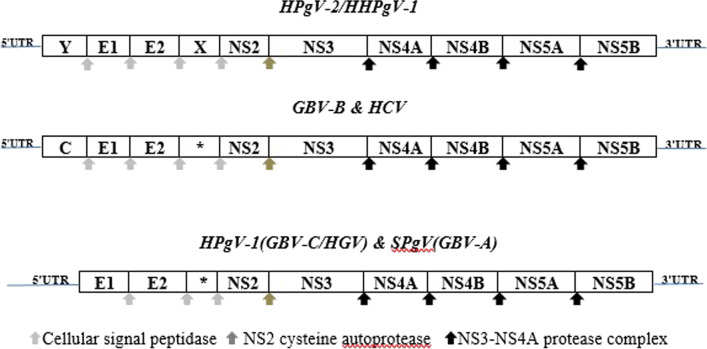
Genome organization of hepaciviruses and pegiviruses. Coding region of a core (C) protein has not been identified for SPgV (GBV-A) or HPgV-1 (GBV-C). Hepegiviruses (HPgV-2/HHPgV-1) encode a protein named Y that is similar in properties and location to HCV & GBV-B. * The predicted sizes of the pore-forming ion channel proteins comparable to the HCV P7 are 21 kDa for SPgV, 6 kDa for HPgV-1 and 13 kDa for GBV-B. The GBV-B 13 kDa protein could be cleaved into P7 and P6 proteins, that the P7 protein, but not the P6 is essential for viability in vivo

HPgV-1 has a 9.4 kb positive polarity RNA (+ ssRNA) genome and its genome organization is like HCV. The genomes of HPgV-1 and HCV comprise 5′ and 3′ non-translated regions (NTRs) and have a long open reading frame (ORF) coding about 3000 amino acids that are co- and post-translationally broken down into structural and non-structural (NS) proteins [28, 52]. The HPgV-1 5′ntr (555 bp) sequence is expected to be larger than the 5′ntr (341nt) of the HCV and internal ribosome entry site (IRES) is used by both viruses that leads to mRNA translation (cap-independent translation), although HPgV-1 IRES has much less activity than that of HCV [52]. While they share common structural elements, The 3′ntr of HPgV-1 (around 300 bp) differs from the HCV one, because it lacks poly-(U) or poly-(A) sequences [53]. Experimentally, translation and protein processing has been proved in HCV; but, HPgV-1 processing is highly focused on assumptions using sequence similarities with HCV. The coding area in HPgV-1 and HCV for structural proteins (Core (C) for HCV and envelope glycoproteins of E1 and E2 for both) is one-third of ORF in the *N*-terminal which are broken down by the signal peptidases of the host cell [54]. HCV, GBV-B, and HHPgV-1/HPgV-2(Y) ORFs encode a core protein upstream of E1. Unlike HCV, HPgV-1 does not contain a sequence for a Core protein but has similar biophysical properties as HCV [55–57]. HPgV-1 encodes E1 and E2 glycoproteins, which probably mediate binding and entry. It has been suggested that HPgV-1 uses the low-density lipoprotein receptor for entrance [58–60]. The open reading frame C-terminus codes the non-structural proteins (*, NS2, NS3, NS4A, NS4B, NS5A, and NS5B). Experimentally, the roles of HPgV-1 non-structural proteins have not been determined, but are expected to be comparable to HCV. Most pegiviruses express small polytopic membrane proteins, analogous to the P7 protein of hepacivirus, immediately after structural proteins. The pore-forming ion channel protein (5.6 kDa) of the HPgV-1 is estimated to be smaller than HCV P7 [61, 62]. HPgV-1 NS2 and NS3 proteins are expected to act as viral proteases. The NS2-NS3 cleavage is performed by NS2 cysteine autoprotease. It is predicted that the NS3 N-terminus has serine protease activity and the C-terminal has NTPase and helicase activities, thus, the residual non-structural proteins are broken down by the NS3 serine protease accompanied by its NS4A co-factor (NS3–NS4A protease complex). NS4B is a nucleotide-binding membrane protein, NS5A is a Zn^2+^—binding phosphoprotein [61, 62] and NS5B is an RNA-dependent RNA polymerase [28, 63, 64] (Fig. 4).

### HPgV-1 genotypes

To date, using phylogenetic analyses and according to the sequence variability of either complete genome or a limited genomic length such as 5'UTR, E1, E2, NS3, and NS5, HPgV-1 has been categorized into seven genotypes and numerous subtypes. These genotypes and subtypes have different geographic distribution patterns [22, 65, 66].

**Fig. 5:**
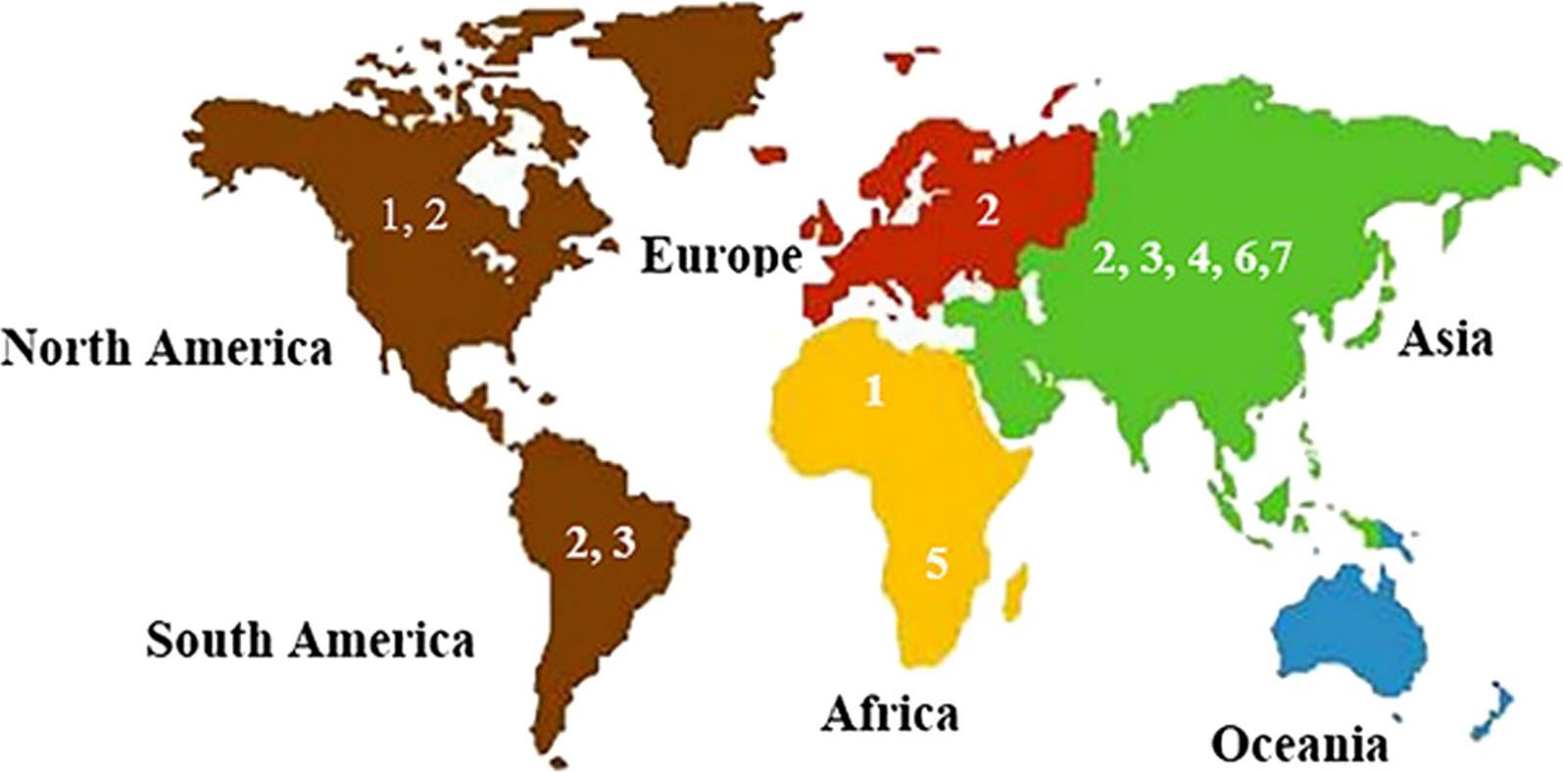
Geographical distribution of different HPgV-1 genotypes

Genotype 1 is mainly found in North America, Africa and has five subtypes [67, 68], genotype 2 (Subclassified 2a and 2b) in North/South America, Europe and Asia [34, 47, 67, 69–71], genotype 3 in South America and Asia [22], genotype 4 in Asia [72], genotype 5 in South Africa [73], genotype 6 is common predominantly in Indonesia [74] and in Yunnan Province, China, the seventh genotype was identified [16, 72] (Fig. 5).

In Asia, HPgV-1 genotypes 2 (35.6%) and 3 (34.7%) are the most common genotypes, although genotypes 7 (10.6%), 1 (5.7%), 4 (5.6%), 6 (3.1%) and 5 (1.1%) have also been reported [47]. In Africa, HPgV-1 genotype 1 (66.2%) is the predominant genotype. Genotype 5 (28.8%) is prevalent in central/east Africa, as well as South Africa. Genotype 2 (5.0%) of HPgV-1 is common in central/west Africa and South Africa [46].

It has been reported that the infection of the same individual with multiple HPgV-1 strains and/ or genotypes is possible [75], and Recombination has also been identified in the HPgV-1 genome [76]. The genotypical diversity of HPgV-1 is therefore vast and could influence viral phenotypes such as interferon sensitivity, cytokine/chemokine expression, cell tropism, and replication [34, 77, 78]. In addition, HPgV-1 genotypic variation may have a function in modulating the development of disease in individuals diagnosed with HIV-1, for instance, in higher T CD4^+^ count and a lower load of HIV-1 [79, 80]. In a 2018 research, Ruegamer et al. found that Human pegivirus type 1 genotype and coreceptor tropism of HIV-1 influence the ability of peptides derived from HPgV-1 E2 protein to inhibit HIV-1 infection [81].

### Disease association

Between 1996 and 1998, various studies were conducted to see if HPgV-1 (HGV/GBV-C) was the reason for acute or chronic hepatitis. There was no epidemiological link between the presence of this novel virus and acute or persistent hepatitis in prospective or controlled retrospective researches [13, 18, 47]. As a result, this virus isn't a hepatitis virus by definition and the word hepatitis G virus (HGV) is fallacious. Similarly, there is no reason to indicate that the surgeon G.B. was diseased by the GBV-C virus [13]. So, neither GBV-C nor HGV define it correctly [3, 82]. There has been no compelling proof to date that HPgV-1 causes any human disease [11, 27]. There have also been multiple attempts to relate HPgV-1 to other diseases, like Sjogren's' syndrome, hepatocellular carcinoma, cryoglobulinemia, lichen planus, and malignant or non-malignant hematological diseases, but no clear link between these illnesses and HPgV-1 infection has been determined [13].

Nonetheless, a correlation between HPgV-1 and better clinical course in patients diagnosed with HIV has been reported; and a reverse link between HPgV-1 and HIV plasma viral load has been demonstrated in some trials [83]. Research also revealed that HPgV-1 coinfection in patients with the Ebola virus is associated with increased survival and lower mortality rates [21].

Contrary to what had been thought earlier, many pieces of evidence have elucidated the lymphotropic nature of HPgV-1 [84]. Related to its replication potential in both T (CD4^+^ and CD8^+^) and B lymphocytes [11] rather than in hepatocytes [48], HPgV-1 viremia has been proposed to be related to an increased risk of non-Hodgkin lymphoma [27, 85].

Some Flaviviridae family viruses, including tick-borne encephalitis virus and West Nile virus, are neurotropic and cause encephalitis [86]. These observations raise the question of whether HPgV may be a causative agent in neuroinfections. In fact, despite significant advances in diagnostics, the etiology of encephalitis remains unknown in many cases. In several case reports done so far, HPgV RNA has been detected in the central nervous system of humans, indicating that the virus can be found in the brain in certain conditions. It is not clear whether encephalitis was caused directly by HPgV-1 in these patients [87–89].

### HPgV-1 and HIV co-infection

In 1998, two major longitudinal pieces of research showed that, in comparison to patients who were consistently negative for HPgV-1 RNA or lacked viremia, the persistence of HPgV-1 viremia in co-infected patients with HPgV-1/HIV was strongly correlated with extended survival [90]. Moreover, a meta-analysis showed that HPgV-1 viremia was correlated with a 2.5-fold decrease in the fatality rate during research that took place ‘late’ (bigger than 5 years) in HIV infection (n = 1294) (Fatality Comparative Risk 0.41; 95% confidence intervals 0.23–0.69) [19]. In several investigations, HPgV-1 viremia has also been correlated with enhanced HIV surrogate indicators like upper CD4^+^ T cell counts, lowered load of HIV, slower progress, and increased antiretroviral therapy response in AIDS-infected patients [18, 83, 91–93]. Furthermore, HPgV-1 viremia is related to the decreased transfer of HIV from mother to child, especially if the baby is infected with HPgV-1 during parturition [94].

Many studies have investigated the effect of the HPgV-1 genotype and subtype on the development of HIV infection [72]. For instance, in a study, the second genotype of HPgV-1 was linked to upper CD4^+^ counts compared to genotype 1 during HIV/HCV/HPgV-1 triple infection [13]. In addition, Muerhoff et al. in 2003 reported that HPgV-1 genotype 2b is associated with higher levels of CD4^+^ cell count, compared to genotype 2a [34, 95, 96]. Therefore, HPgV-1 genotype may have a differential effect on the development of HIV disease; nevertheless, more study is needed in larger samples with several circulating genotypes of HPgV-1.

Multiple studies in vivo and in vitro have indicated that HPgV-1 infection can both directly interfere with HIV replication and influence cellular elements that contribute to the HIV life cycle. E1, E2, NS3, and NS5A are HPgV-1 proteins that prevent HIV replication. The results of HPgV-1 infection on HIV-positive individuals are listed below [24].

1. The course of HIV disease is correlated with a shift in cytokine patterns from T helper one to T helper two. NS5A stimulates cytokines of Th1 (IL-12, IL-2 and IFN-γ) as well as decreases the expression of Th2 cytokines (IL-10, IL-4 and IL-13), so HIV patients with HPgV-1 viremia, have more stable Th1 cytokine levels compared to HIV mono-infected patients [97, 98].
2. NS5A downregulates the expression of CD4, CXCR4 and induces SDF-1, the soluble CXCR4 ligand [99–101].
3. Circulating CD80^+^ plasmocytoid dendritic cells (CD80^+^pDCs) are an important source of Th1 cytokines, which are enhanced in number upon HIV/HPgV-1co-infection [102].
4. HPgV-1 also increases the level of interferons expression [103].
5. In addition, the release of C–C motif Chemokine Receptor five (CCR5) soluble ligands (MIP-1a, MIP-1β, and RANTES), are increased in co-infection with HPgV-1, resulting in reduced CCR5 (the other HIV coreceptor) surface expression [100, 104].
6. The interaction of both HPgV-1 E1 and E2 with the fusion protein of the HIV-1 glycoprotein 41 (gp41) and HPgV-1 E2 with HIV-1 glycoprotein 120 (gp120) V3 loop results in direct inhibition of HIV entry [80, 105–109].
7. Additionally, HPgV-1 E2 protein contains a Tyk2 interacting motif that inhibits NK cell IL-12-mediated IFNγ release, resulting in reduced T cell activation (lower proportion of T lymphocytes with CD38 expression), proliferation, and function [27, 110].
8. HPgV-1 E2 Antibodies have been shown to neutralize HIV-1 infection through viral attachment inhibition [111].
9. Co-infection with HPgV-1 declines the expression of Fas on B and T lymphocytes, hence decreasing apoptosis caused by Fas [80, 84].
10. Lastly, HIV replication in a T lymphocyte is blocked by the HPgV-1 NS3 serine protease without reducing CXCR4 coreceptor or CD4 expression., introducing an independent mechanism of entry followed through proteolytic cleavage of the unknown target(s) (viral or cellular proteins required for HIV efficient replication) [112] and the infected T lymphocytes may be biased toward a T helper one phenotype [98].

**Fig. 6:**
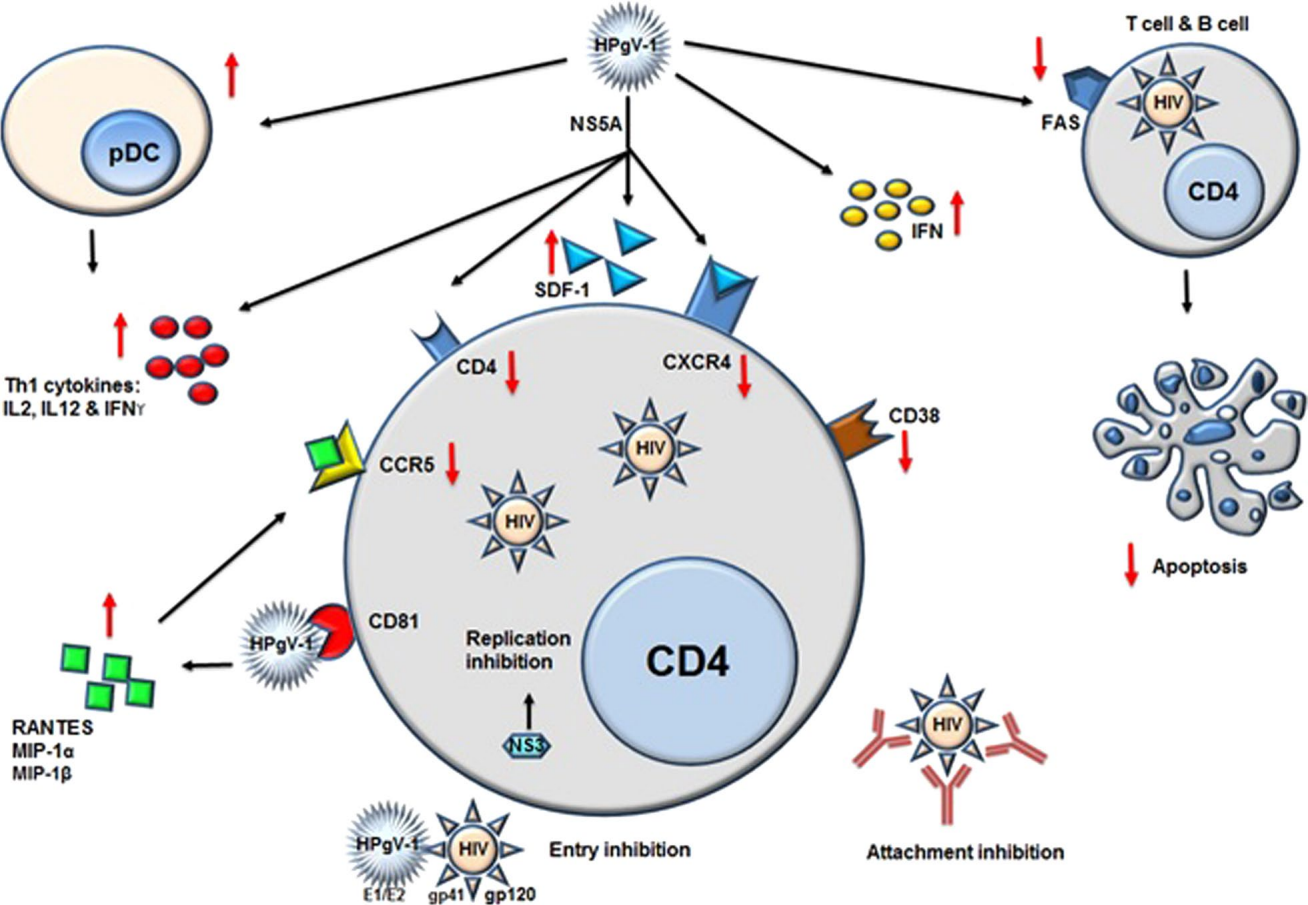
proposed mechanisms of HIV-1 inhibition during the co-infection HPgV-1/HIV. *Ab* antibody, *SDF-1* stroma-derived factor-1, *CCR* c–c chemokine receptor, *RANTES* regulated on activation, normal T-cell expressed and secreted, *pDC* Plasmocytoid dendritic cell

In summary, the HPgV-1 can interact with HIV replication and slow down the development of the disease by reducing surface expression of CXCR4 and CCR5 and by inducing the release of soluble ligands for CCR5 (MIP-1a, MIP-1b, and RANTES) and CXCR4 (SDF-1). Moreover, HPgV-1 decreases activation, apoptosis, and proliferation of T cells. This virus also increases interferons expression, stimulates pDCs, and enhances cytokines of Th1, resulting in improved non-specific immune responses. Together, these consequences would possibly restrict the spread of HIV and reduce the progression of the diseases (Fig. 6).

### HPgV-1 and HCV co-infection

Co-infection with HPgV-1 and HCV is common, and HPgV-1 is linked to HCV endemicity. However, HPgV-1 does not appear to have any adverse effect on the progression of HCV chronic liver disease. It also does not influence the hepatic histology, transaminase rates, or antiviral treatment response [113]. Boodram et al. recorded an increased level of HPgV-1 clearance in HIV-negative IDUs (Injection drug users) with HCV infection [114]. Of note, HPgV-1/HCV co-infection rates differ significantly by geographic region. Among Asian countries, co-infection with HPgV-1/HCV has been seen to be more widespread [115]. While there have been few studies on the co-infection between HBV and HPgV-1, it has been shown that HPgV-1 has no impact on HBV-DNA rates or development of infection [13].

### HIV/HCV/HPgV-1 triple infection

Although HPgV-1 does not affect the outcome of HCV mono-infection, HIV has a major adverse effect on the progression of HCV infection. For instance, during HIV/HCV co-infection, HCV RNA amounts are greatly increased compared to HCV mono-infection [116]. In addition, the risk of liver fibrosis, cirrhosis, and end-stage liver disease, which are related to HCV disease, are increased after coinfection with HIV/HCV [117]. It is especially important since HCV has been identified as a significant reason for death in people diseased with HIV [118]. As with HCV, HPgV-1 is also susceptible to interferon's antiviral impact [119]. It is therefore essential to investigate how the HIV/HCV/HPgV-1 triple infection can affect the development of HIV and liver diseases. Besides, the clearance of HPgV-1 RNA may have a negative effect on HIV-related results, especially in patients receiving IFN therapy. Barbosa et al. have reported a lower ratio of hepatic lesions in infected HCV/HIV/HPgV-1 patients compared to co-infected HCV/HIV cases [120].

### HPgV-2 VS HPgV-1

HPgV-2 and HPgV-1 are blood-borne, lymphotropic, but not a hepatotropic viruses [10, 11, 34, 121], thereby people with hemophilia and persons who Inject drugs with prolonged exposure to the blood and infected with HIV-1 and/or HCV are most often contaminated with HPgV-2 and HPgV-1 [122]. Multiple pieces of researches have shown the difference between HPgV-2 and HPgV-1 in incidence, spread, and phylogeny. The overall prevalence of HPgV-2 (1.33%) in all populations studied is lower than HPgV-1 (35%) [122]. While co-detection of RNA and E2 antibodies in HPgV-1 infection is rare (5.88%), antibodies against E2 are found, like HCV, in most HPgV-2 viremic persons (92.86%) [123]. It has been shown that approximately 30% of HPgV-1 and HPgV-2 infections are persistent and the other 70% of infected subjects clear viremia during disease [38, 122, 124]. Findings indicate that, like HPgV-1, HPgV-2 coinfection with HCV is unable to exacerbate liver damage or influence HCV replication. Lastly, HPgV-2 infection has not been related to any specific symptoms or changes in the progression of HIV disease [122].

### Immunity

HPgV-1 exposure leads to acute infection after an incubation time predicted to be 14–20 days. This infection can move into a recovery phase, with the disappearing of serum HGV RNA and the appearance of anti-E2 antibodies, which seem to provide partial reinfection defense [27] or persistent infection with serum HGV RNA, which is constantly observable [125]. Though HPgV-1 viremia may remain for ages, most immunocompetent people contaminated with HPgV-1 clear viremia during 2 years following infection [126].

E-protein is an essential agent of HPgV-1 (engaged in virus adhesion and fusion with host cells) and is considered an appropriate candidate for the development of anti-HPgV-1 antibodies. When the proportion of E2 antibody positive blood donors is compared to those with HPgV-1 viremia, it indicates that nearly 75% of HPgV-1 infections are automatically cleared by the body, especially in individuals with immune competence [5, 13, 18]. The prevalence of E2 Ab in blood donors is around two- to six-fold greater than HPgV-1 RNA [90].

Simultaneous identification of E2 antibody and viral RNA appears rare and only happens for a short period [44]. The HPgV-1 viremia and anti-E2 antibody coexistence occur just before clearance, possibly in the shape of an immunocomplex [127]. In multicenter research on HIV-infected persons, 1.8% of HPgV-1 viremia patients had measurable anti-E2, whereas 75% of HPgV-1 RNA negative cases had E2 antibodies [90].

While anti-E2 is an indicator of the previous infection, its identification can be lost over time [24]. This phenomenon usually occurs faster among HIV-infected people and therefore it may be hard to accurately calculate the true occurrence of HPgV-1 infection for them [128]. There is some indication that patients and children contaminated with HIV will clear HPgV-1 more slowly than people with intact immune systems [129] and the HPgV-1 viremia is far more prevalent in people with immunosuppression than in healthy blood donors [130].

### Detection

Diagnosis of HPgV-1 is usually done via ELISA and nested reverse transcription PCR (RT-PCR). The two tests mentioned above are focused on the detection of distinct viral markers in biological samples. The existence of HPgV-1 RNA is dependent on nucleic acid amplification and quantification, performed by real-time RT-PCR, and ELISA identifies E2-protein antibodies [27].

Given the prevalence of HPgV-1 disease in common people, and its positive impact on people infected with HIV, the development of commercial diagnostic tests for detection of E2 antibodies or HPgV-1 viremia, which are currently unavailable, seems necessary. As described in previous sections, HPgV-1 seems to slow the progression of HIV disease and can contribute to the control of T lymphocyte balance in vivo. In this regard and to gain a well understanding of the status of HPgV-1 infection and thus allow a better investigation of the influence of HPgV-1 on other viral infections, it is extremely important to establish active or prior HPgV-1 infection assays [24, 131, 132].

## Conclusion

Like all viruses, HPgV-1 relies on the host for replication. Until now, not only there has been found no compelling connection between HPgV-1 and any human illness but also it tends to be defensive against HIV infection and other illnesses, emphasizing a mutually advantageous symbiotic relationship. Epidemiologic studies have identified a relationship between HPgV-1 co-infection and lower fatality rates among HIV- diseased individuals. More analysis of the interactions between HPgV-1 and HIV has the potential to open up new treatment options for HIV disease later.

On the other hand, the anti-inflammatory properties of HPgV-1 tend to be favorable in managing some immune-mediated diseases. As an example, the impact of HPgV-1 on the host immune system function may lead to the reduction in the processes for immune surveillance observed in non-Hodgkin lymphoma [27]. An important part that still needs to be clarified is the mechanisms through which HPgV-1 infects various types of blood cells and survives in humans. Further studies should be carried out to better understanding the processes of HPgV-1 persistence and clearance in humans. Given that human pegiviruses are phylogenetically a close relative of HCV, HPgV-1 may be used as a replacement model for HCV infection to clarify pathogenesis, immunology, and HCV persistence in humans, as well as to enhance the possible production of new vaccines and immunotherapies [133].

## Data Availability

Not applicable***.***

## Abbreviations

HPgV-1: Human pegivirus type 1
ssRNA: Single-stranded RNA
HCV: Hepatitis C virus
T cell: Thymus cell lymphocytes
Bcell: A lymphocyte derived from bone marrow
CD4+: Cluster of differentiation 4
CD8+: Cluster of differentiation 8
HIV-1: Human immunodeficiency virus type1
Pegivirus: Persistent GB viruses
ORF: Open reading frame
E2: Envelope glycoprotein E2
ELISA: Enzyme-linked immunosorbent assay
RT-PCR: Reverse transcription polymerase chain reaction
G.B: G. Barker
GBV-A, B, C, D: GB virus type A, B, C, D
SPgV: Simian pegivirus
BPgV: Bat pegivirus
CPZ: Chimpanzees
RS: Representive species
HHpgV-1: Human hepegivirus type1
HPgV-2: Human pegivirus two
mRNA: Messenger RiboNucleic acid
ER: Endoplasmic reticulum
NTR: Non-translated regions
NS: Non-structural
IRES: Internal ribosome entry site
C: Core
LDLR: Low density lipoprotein receptor
P7: Pore-forming ion channel protein
5'UTR: Un-translated region
AIDS: Acquired immune deficiency syndrome
Th1: T helper 1
IL-2: Interleukin 2
IFN-γ: Interferon gamma
CXCR4: C–X–C motif chemokine receptor type 4
CD80+pDC: CD80+ plasmocytoid dendritic cell
CCR5: C–C motif chemokine receptor
MIP-1a: Macrophage inflammatory protein 1-α
MIP-1β: Macrophage inflammatory protein 1-β
RANTES: Regulated on activation, normal T-cell expressed and secreted
SDF-1: Stroma-derived factor-1
gp41: Glycoprotein 41
gp120: Glycoprotein 120
V3 loop: Variable loop 3
Tyk2: Tyrosine kinase 2
NK cell: Natural killer cell
CD38: Cluster of differentiation 38
Ab: Antibody
IDUs: Injection drug users
DV2: Dengue virus serotype 2
JEV: Japanese encephalitis virus
WNV: West nile virus
YFV: Yellow fever virus
TBEV: Tick-borne encephalitis virus
